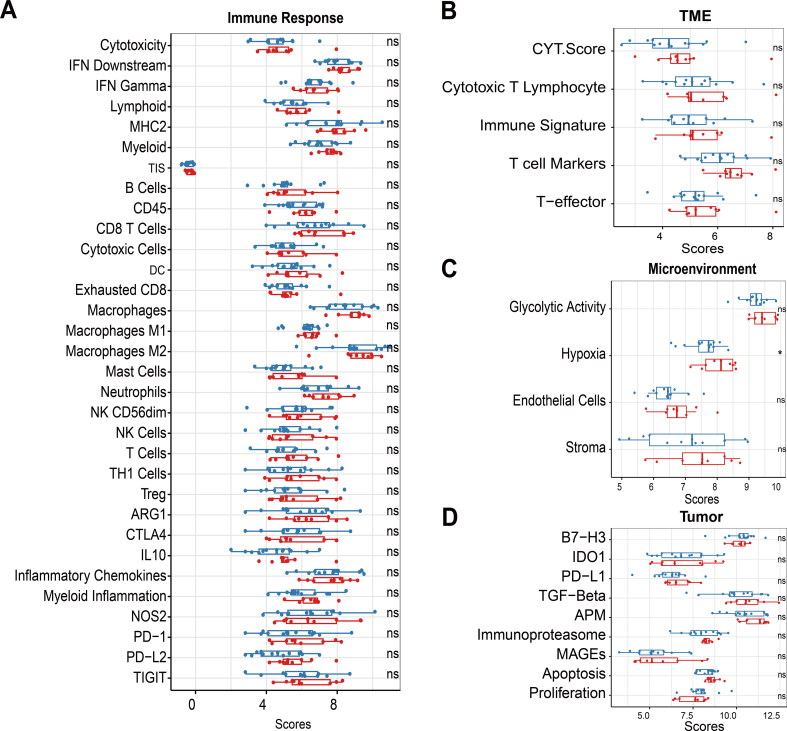# Alterations of the tumor microenvironment (TME) by exploratory gene expression analysis in recurrent glioblastoma following apatinib in combination with temozolomide

**DOI:** 10.1016/j.bbrep.2025.102364

**Published:** 2025-12-01

**Authors:** Jingjing Ge, Cheng Li, Fengjun Xue, Chi Zhao, Chenchen Kong, Shaopei Qi, Qianqian Duan, Qin Zhang, Junping Zhang

**Affiliations:** a Department of Neurosurgery, Sanbo Brain Hospital, Capital Medical University, Beijing, China; b Department of Neuro-Oncology, Sanbo Brain Hospital, Capital Medical University, Beijing, China; c The Medical Department, State Key Laboratory of Neurology and Oncology Drug Development, Jiangsu Simcere Pharmaceutical Co.,Ltd., Jiangsu Simcere Diagnostics Co.,Ltd., Jiangsu Province, China

**Keywords:** Recurrent glioblastoma, Apatinib, Temozolomide, Tumor microenvironmental, Gene expression

## Abstract

Apatinib in combination with temozolomide (TMZ) has achieved reasonable clinical efficacy in the treatment of recurrent glioblastoma (rGBM), however, there are currently no clear biomarkers related to clinical efficacy or prognosis. Our retrospective study was to investigate tumor microenvironment (TME) features at the gene expression level that are associated with response and long survival benefit of rGBM treated with apatinib and TMZ. We enrolled 22 rGBMs treated with apatinib in combination with TMZ and collected their tissue samples for RNA transcriptome analysis by the Nanostring nCounter platform. The response group had 40 differentially expressed genes compared to the non-response group, with significantly up-regulated expression of genes related to endothelial cells and apoptosis. Enrichment analysis revealed that signaling pathways related to cell proliferation were down-regulated in the response group. In terms of prognosis, there were 16 differential expressed genes in the long-survival benefit group compared with the short-survival benefit group, and four tumor progression-associated genes were also down-regulated in response group expression. Hypoxia related-genes was significantly up-regulated in the long survival benefit group. Enrichment analysis showed that genes related to cell proliferation were also down-regulated in the long-survival benefit group, while the expression of signaling pathway genes related to cell activation, and immune response was significantly up-regulated. Our study suggests that the combination of apatinib and TMZ may potentially provide clinical benefits in treating rGBM by modulating genes associated with cell proliferation, promoting apoptosis, regulating hypoxia, and enhancing immune response within the tumor microenvironment.

## Highlights

• Apatinib combined with TMZ modulates the TME in recurrent GBM.
• Therapy reshapes the TME by suppressing cell proliferation and enhancing immunity.
• Long survival GBM correlates with hypoxia gene and immune response activation.

## 1 Introduction

Glioblastoma (GBM) is the most prevalent and highly malignant primary central nervous system tumor that has a global incidence of 0.59–3.69 cases per 100,000 [1]. The standard treatment for glioblastoma is maximal surgical resection followed by concomitant radiotherapy and temozolomide chemotherapy, but patient's survival still remains poor, with a 1-year survival rate of 40.6 % and a five-year survival rate of only 5.6 % [2]. The effective treatment strategies for progressed or recurrence glioblastoma (rGBM) are limited. Apatinib is an oral small molecule inhibitor that selectively targets vascular endothelial growth factor receptor-2 (VEGFR2) and could inhibit angiogenesis [3]. Previous studies have reported significant efficacy of apatinib monotherapy or combination therapy in the treatment of many solid tumors, including non-small cell lung cancer [4] and hepatocellular carcinoma [5]. Currently, the combination of apatinib and temozolomide (TMZ) in the treatment of rGBM has also yielded certain clinical benefits. The objective response rate ranges from 24 % to 45 %, with a disease control rate of 82 %–90 %. The median progression-free survival (PFS) is 4–6 months, and the median overall survival (OS) is 8–9 months [[6], [7], [8]].

However, there are still some patients who do not benefit from treatment with apatinib combined with TMZ, so screening for effective efficacy markers is clinically important for patients with rGBM. Previous cell assay has shown that apatinib could inhibit glioma cell growth and metastasis as well as promoted the anti-tumor activity of temozolomide [9]. However, no clinical studies have reported the mechanism of therapeutic response of apatinib in combination with TMZ in rGBM. Notably, there are many studies about bevacizumab, another anti-angiogenic drug with a long history of clinical application. Bevacizumab combinated with TMZ could inhibit IDH mutant GBM cell lines by up-regulating extracellular matrix and immune response related signaling pathways as well as down-regulating signaling pathways related to cell proliferation [10]. Bevacizumab could restore the immune-supportive microenvironment by decreasing PD-1 expression in immune-infiltrating lymphocytes (TILs), as well as decreasing Tregs, and this modulation persisted even in relapsed patients [11]. Therefore, we hypothesize that apatinib combined with TMZ may affect the tumor microenvironment of rGBM, which is related to the difference in efficacy after treatment of these patients.

The present study retrospectively enrolled 22 patients who received apatinib in combination with TMZ therapy for rGBM after progression on standard radiochemotherapy therapy. The tissue samples from rGBMs were collected for transcriptomic analysis using a tumor microenvironment related 770 genes panel. The goal of this analysis was to uncover the regulatory mechanism behind the treatment of rGBM using a combination of apatinib and TMZ in terms of TME, and aimed to identify potential markers that could be used to evaluate the effectiveness of the treatment or predict the prognosis of rGBMs.

## 2 Material and methods

### 2.1 Patient selection

22 rGBMs were enrolled with residual tissue specimens who received treatment with apatinib combined with temozolomide from the cohort we previously published [6]. We collected their pre-treatment of apatinib tissue samples for RNA transcriptome sequencing. The study also included 6 patients who received standard radiotherapy combined with TMZ and experienced recurrence. Their primary samples and paired post-treatment recurrence samples were collected for RNA transcriptome sequencing. The study design was shown in Fig. 1.

**Fig. 1:**
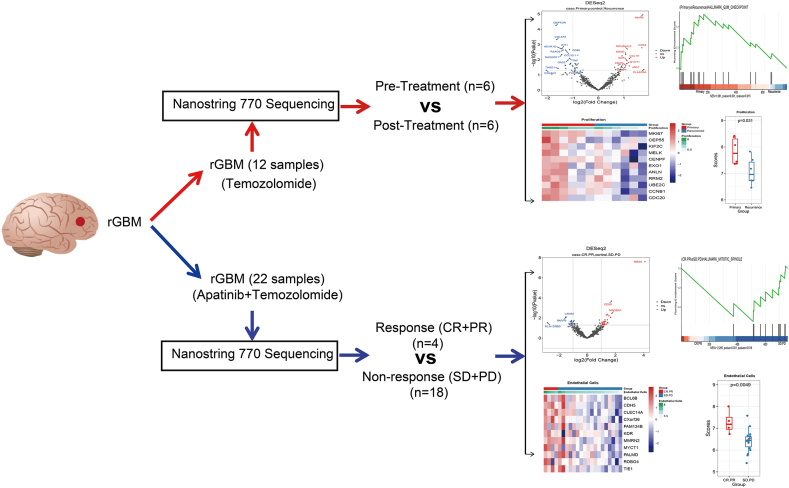
The study design flowchart.

The study was registered on ClinicalTrails (NCT04814329) and approved by the institutional ethics committee of Sanbo Brain Hospital, Capital Medical University (SBNK-YJ-2021-010-02), and informed consent was also obtained from the patients.

### 2.2 Outcomes

The clinical responses was classified based on the Response Assessment in Neuro-Oncology (RANO) criteria [12] as complete response (CR), partial response (PR), stable disease (SD), or progression of disease (PD). Progression-free survival (PFS) refers to the time from enrollment to the first recorded disease progression or death from any cause. In prognosis analysis, long survival benefit is defined as a PFS≥4.2 months, and short survival benefit is defined as a PFS<4.2 months. This PFS threshold was primarily referenced from a Phase III study of bevacizumab combined with chemotherapy for rGBM [13].

### 2.3 Nanostring-panel RNA sequencing

Total RNA was isolated from FFPE slices using the Qiagen RNeasy FFPE Kit, followed by hybridization of 100 ng RNA to a version of the NanoString PanCancer code set for reading on the nCounter platform. We employed a 770-gene panel (The nCounter PanCancer IO 360™ Panel) to scrutinize the gene expression profile, with a specific focus on genes implicated in the intricate interplay between different immune cell types, common checkpoint inhibitors, CT antigens, and genes covering both the adaptive and innate immune response in cancer. All sample handling and testing are based on standard operating recommendations [14]. For each sample, quality control indicators, including the Imaging QC, Binding Density QC, Positive Control Linearity QC, Positive Control Limit of Detection QC, Positive normalization factor, and Content normalization factor, were then calculated. Samples that passed the quality control were included in the subsequent analysis. The NanoStringNorm package in R was used to normalize raw data [15]. The housekeeping genes were employed to normalize the expression values, as recommended by the manufacturer, using nSolver 2.6 software.

### 2.4 DEGs and functional enrichment analysis

Differentially expressed genes (DEGs) were selected through the “DEseq2” [16]software package, with log2 |fold change| > 1 and false discovery rate <0.05. Heatmaps of differentially expressed genes were created using the “ComplexHeatmap” package [17]. We then used Gene Ontology (GO) and Kyoto Encyclopedia of Genes and Genomes (KEGG) analysis to analyze the possible biological processes in which the overlapping DEGs are involved. Also, genomic enrichment analysis (GSEA) analysis was used to further explore gene expression differences in each group.

### 2.5 Estimation of tumor immune microenvironment and signatures

According to the manufacturer's specification, the genes were divided into 14 immune cell types: T cells, B cells, mast cells (MCs), dendritic cells, macrophages, neutrophils, cytotoxic cells, exhausted CD8, NK-CD56, CD8 T, CD45, Th1, NK, and Treg cells [[18], [19], [20]]. Signatures of IFN-γ signature, GEP score, T cell markers and chemokines were achieved using a specific set of genes with relevant biological function, respectively [21,22]. The GEP score was calculated as a weighted linear average of the constituent genes while the remaining signatures were calculated as the arithmetic means of the corresponding gene [21]. Furthermore, we studied previously published gene sets and their metagene scores using the previously described methods [[23], [24], [25], [26], [27], [28]].

### 2.6 Protein-protein interaction (PPI) network analysis

To test the interactions of the identified DEGs, we performed protein–protein interaction (PPI) network analysis. All PPI analyses were conducted using the Search Tool for the Retrieval of Interacting Genes (STRING) database.

### 2.7 Statistical analysis

Statistical analyses and graph illustration were performed using GraphPad Prism 6.0 (GraphPad Software, San Diego, California, USA), and R software version 4.1.3 (R Foundation for Statistical Computing, https://www.R-project.org/). Statistical comparisons were performed using unpaired two-sided Student *t*-test or Mann–Whitney *U* test, according to the variances. P value < 0.05 was used as a significant threshold in the remaining statistical analysis.

## 3 Results

### 3.1 Clinical characteristics of enrolled patients

In apatinib group, all patients received oral apatinib 500 mg once daily in combination with temozolomide. Temozolomide was administered at 200 mg/m^2^/d according to the standard 5/28 days regimen for patients who had not previously received temozolomide. Patients who experienced a relapse following the standard 5/28 temozolomide schedule received continuous daily temozolomide (50 mg/m^2^/d). One treatment cycle was defined as 28 days (4 weeks). Patients continued treatment until they experienced disease progression or unacceptable toxicity [6]. According to the efficacy assessment, there were 1 case CR, 3 cases PR, 6 cases SD, 12 cases PD. Detailed 22 rGBMs information is shown in Table 1. The six patients with standard radiochemotherapy (radiotherapy combined with temozolomide chemotherapy) whose therapeutic clinical information is presented in Supplementary Table S1.

**Table 1:** The clinical information of 22 recurrent glioblastoma patients.

Characteristics		Patients (n = 22)
Age (year)	Median (range)	55 (28–70)
Sex, n (%)	Male	16 (73 %)
	Famale	6 (27 %)
Initial KPS, n(%)	90–100	10 (45 %)
	70–80	5 (23 %)
	50–60	7 (32 %)
≥2 recurrence before enrollment	Yes	5 (23 %)
	No	17 (77 %)
Best Response (RANO Criterial)	CR	1 (5 %)
	PR	3 (14 %)
	SD	6 (27 %)
	PD	12 (55 %)

### 3.2 Effect of standard radiochemotherapy on the TME of GBM

Since all patients relapsed after standard chemoradiotherapy, we first investigated the effects of standard therapy on the immune microenvironment. 6 GBM who received standard radiotherapy combined with TMZ and experienced recurrence and their primary samples and paired post-treatment recurrence samples were collected for RNA sequencing.

There were 32 significantly differentially expressed genes (P < 0.05) in primary samples compared to the recurrent samples (Supplemental Table S2). Notably, the matrix metalloproteinase-9 (MMP9) gene was significantly lower expressed in the recurrent samples, while the *DEPTOR* gene, encoding a DEP-domain containing mTOR-interacting protein, was significantly up-regulated in the recurrent samples (P < 0.01; P < 0.01; Fig. 2A). The previous literature has reported a trend of down-regulation of the *MMP9* gene at both mRNA and protein expression levels in glioblastoma patients treated with radiotherapy combined with TMZ [29]. Moreover, low-expression of MMP9 was a favorable prognostic factor for primary glioblastoma, and it was thought that the glioblastoma with MMP9 low-expression would benefit more from radiotherapy combined with TMZ chemotherapy than radiotherapy alone [30]. Expression of genes associated with proliferation was significantly lower in recurrent samples (P = 0.031; Fig. 2B). Immune cell infiltration analysis showed that the number of M1 macrophages cells was significantly higher in the recurrent samples (P = 0.041, Fig. 2C). In addition, the fragment crystallizable receptors (FCR) score, including *FCGR1A*, *FCGR2A*, *FCGR3A/B*, were significantly higher in the relapse samples than in the primary samples (P = 0.031; Fig. 2D). FcRs are being expressed in immune cells, bind to the Fc part of immunoglobulin, and regulate the interactions between innate and adaptive immune response [31]. In vitro experiments have shown that FcγR effectors play an important role in toll-like receptor agonist-coupled monoclonal antibody drug activation of intratumoral antigen presenting cell (APC) to deliver antigens to T cells and exert durable antitumor effects [32]. However, there were no significantly differences in the tumor, microenvironment and TME signatures between primary and recurrent samples (Supplemental Fig. S1). The recurrent samples contained fewer CD8 T cells and more Treg cells, suggesting that patients with recurrence were in an immune-exhausted state. The results of Gene Set Enrichment analysis (GSEA) showed that only G2M-checkpoint pathway genes and E2F-targets pathway genes were significantly enriched in primary samples (Fig. 2E and F). These results suggest that standard treatment has reduced the expression of genes associated with tumor proliferation and up-regulated the expression of some genes associated with the immune response in rGBM.

**Fig. 2:**
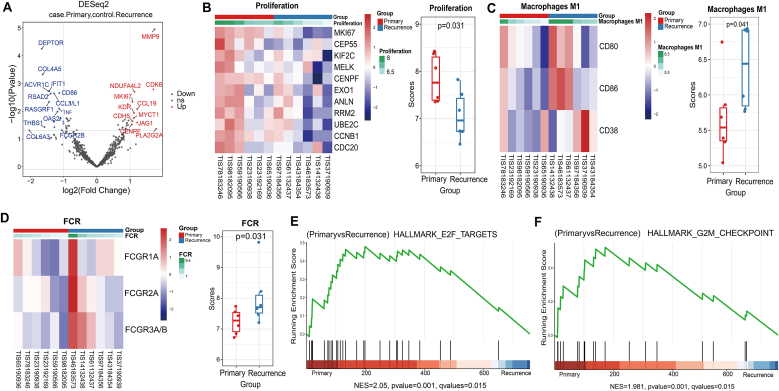
**Characterization of the tumor microenvironment before (Red) and after (Blue) standard radiochemotherapy treatment of GBM.** (A) Volcano plot of differentially expressed genes between primary and recurrent samples. X-axis: log2FC; Y-axis: log10 p-value. Red: upregulated, Blue: downregulated. Selection criteria: log2 |fold change| > 1 and false discovery rate <0.05; (B) Comparison of differential expression of proliferation-associated genes and its score between primary and recurrent samples; (C) Comparison of differential expression of M1-type macrophage and its score between primary and recurrent samples; (D) Comparison of differential expression of the fragment crystallizable receptors (FCR) related genes and its score between primary and recurrent samples; (E–F) Genomic enrichment analysis (GSEA) between primary and recurrent samples. X-axis: Genes ranked by differential expression; Y-axis: Running Enrichment Score; The green curve depicts the running enrichment score; Bar height indicates Normalized Enrichment Score.

### 3.3 TME features analysis of rGBMs treated with apatinib in combination with TMZ

In order to explore the possible molecular mechanisms underlying the efficacy response of apatinib combined with TMZ in the treatment of rGBM, and to screen the molecular markers that effectively predict the efficacy of apatinib combined with temozolomide for rGBM. We categorized 22 patients into a response group (1 CR+3 PR) and a non-response group (6 SD + 12 PD). The response group demonstrated a prolonged PFS (hazard ratio [HR] = 0.162, 95 % confidence interval [CI]: 0.0357–0.7308, Supplemental Fig. S2A). The pre-treatment surgical tissue samples were collected to undergo TME gene expression analysis. Differential gene expression analysis showed that there were 27 up-regulated genes and 13 down-regulated genes in the response group compared to the non-response group (Fig. 3A, Supplemental Table S3). The response group has higher levels of immune cells such as CD8 T cells, DC cells, and neutrophils. However, there was no statistical significant differential expression of immune cells between the two groups (Supplemental Fig. S3A). We found that the expression of endothelial factor-related genes in the microenvironment signatures and apoptosis-related genes in the tumor signatures were significantly up-regulated in the response group (P = 0.0049; P = 0.0064; Fig. 3B and C). The tumor immune microenvironment related indicators (immune cell status, immune score) were not different between the two groups (Supplemental Fig. S3B–S3D). GSEA and GO enrichment analyses revealed that cell cycling or regulation of cell proliferation pathway genes, such as mitotic-spindle, E2F-targets, cellular response to peptide and chromosome segregation, were significantly down-regulated in the response group (Fig. 3D–G). These results suggest that the treatment of rGBM with apatinib in combination with TMZ may promotes apoptosis by down-regulating of the expression of genes involved in cell proliferation related pathways.

**Fig. 3:**
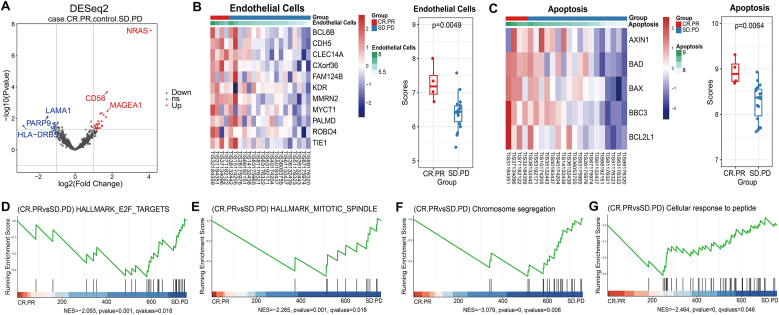
**Characterization of the tumor microenvironment between the response (Red) and non-response (Blue) rGBMs treated with apatinib in combination with TMZ.** (A) Volcano plot of differentially expressed genes between treatment response and non-response groups. X-axis: log2FC; Y-axis: log10 p-value. Red: upregulated, Blue: downregulated. Selection criteria: log2 |fold change| > 1 and false discovery rate <0.05; (B) Comparison of differential expression of endothelial cell associated genes and its score between primary and recurrent samples; (C) Comparison of differential expression of apoptosis-associated genes and its score between primary and recurrent samples; (D–G) Genomic enrichment analysis (GSEA) between response and non-response groups. X-axis: Genes ranked by differential expression; Y-axis: Running Enrichment Score; The green curve depicts the running enrichment score; Bar height indicates Normalized Enrichment Score.

### 3.4 Exploration of biomarkers associated with long survival benefit of apatinib combinated with temozolomide therapy for rGBM

Earlier studies have demonstrated the clinical efficacy of bevacizumab monotherapy or combination therapy in the treatment of rGBM. Based on the results of the EORTC 26101 study, bevacizumab in combination with lomustine significantly prolonged the mPFS (4.2 months vs 1.5 months, HR = 0.49, 95 % CI: 0.39–0.61; P < 0.001) of rGBMs compared with lomustine monotherapy [13]. In addition, the mPFS of 31 rGBM treated with apatinib in combination with temozolomide in our prior clinical study was 4.9 months [6]. The mPFS of the 22 patients enrolled in this study was 3.0 months (95 %CI: 2.7–6.2 months, Supplemental Fig. S2B). Wang et al. [7] conducted a study involving 20 patients with recurrent GBM treated with apatinib in combination with TMZ, reporting a mPFS of 6 months. Yao et al. [8] reported a mPFS of 4 months in a study of 18 patients with recurrent high-grade glioma treated with the same regimen.

Given the comparable mechanisms of action between lomustine plus bevacizumab and apatinib plus TMZ, along with the consistency of median PFS observed in previous studies of apatinib-based therapy for recurrent GBM. And the EORTC 26101 study was a large-sample randomized controlled trial. We decided to choose 4.2 months as the threshold of clinical survival benefit to explore potential biomarkers of long survival benefit associated with apatinib combined with temozolomide for rGBM.

Enrolled rGBMs were categorized into long-survival (PFS ≥4.2 months) and short-survival (PFS <4.2 months) groups, with a 9:13 ratio (Supplemental Fig. S2C). A comparison of clinical characteristics, including age and gender, between the long-survival group (n = 9) and the short-survival group (n = 13) revealed no statistically significant differences between the two groups (Supplemental Table S4).

Tissue samples were collected for gene expression analysis to compare tumor microenvironment profile between the groups and aimed to clarify immune response patterns and identify therapy targets. Differential gene expression analysis showed that there were 16 differentially expressed genes between the two groups (Fig. 4A, Supplemental Fig. S4), of which 4 down-regulated genes (*HELLS, ANLN, LAMA1, UBE2C*) were also low expression in the treatment response group (Fig. 4B). Long-term survivors tend to be in an immunologically active state, with higher levels of B cells, T cells, neutrophils, and DC cells. There were no significant differences between the long and short survival benefit groups in immune cell expression, and tumor signatures as well as TME signatures analyses (Supplemental Fig. S5), but we found that hypoxia-associated genes in the microenvironment signature were significantly up-regulated in expression in the long survival benefit group (P = 0.042; Fig. 4C). GSEA and GO enrichment analyses showed that the expression of genes of signaling pathways related to cell proliferation (mitotic cell cycle, E2F_targets) or regulation of cell proliferation (regulation of cell cycle, DNA metabolic process) was down-regulated in the long survival benefit group, which was concordant with the treatment-responsive group, while the expression of signaling pathway genes related to cell activation, and immune response (response to external biotic stimulus, immune response) was up-regulated (Fig. 4D and E). These results suggest that the treatment of rGBM with apatinib in combination with temozolomide may result in a long-term survival benefit by modulating the down-regulation of genes associated with cell proliferation and promoting the up-regulation of genes associated with immune response.

**Fig. 4:**
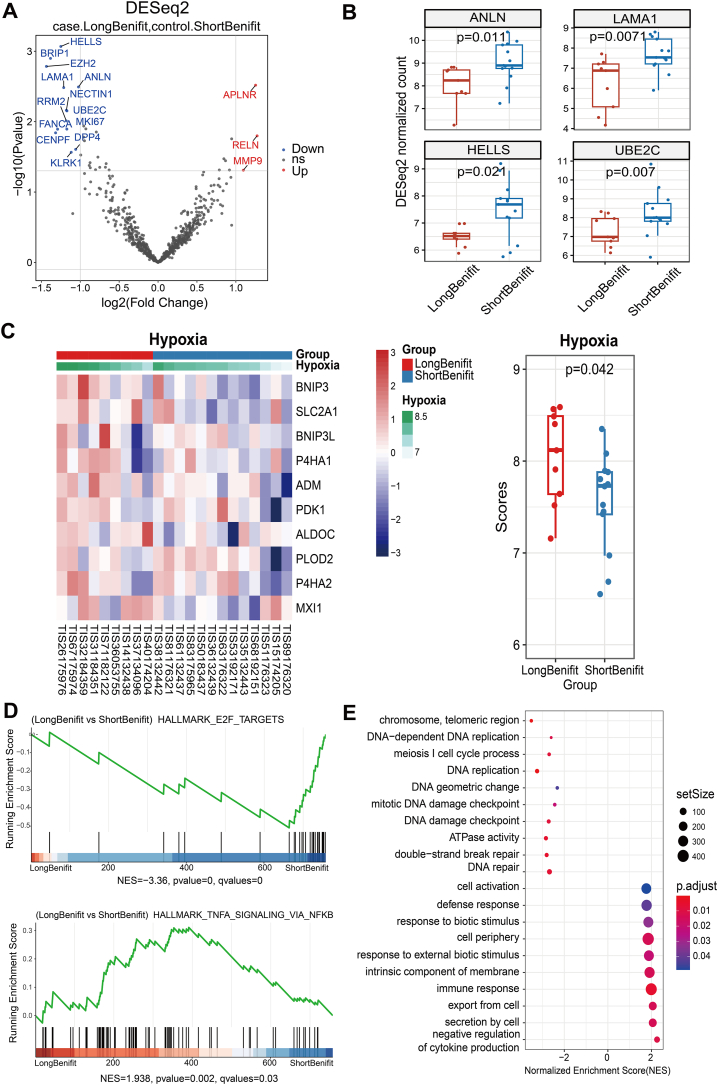
**Characterization of the tumor microenvironment between the long-survival benefit (Red) and short-survival benefit (Blue) groups of rGBMs treated with apatinib in combination with TMZ.** (A) Volcano plot of differentially expressed genes between long-survival benefit and short-survival benefit groups. X-axis: log2FC; Y-axis: log10 p-value. Red: upregulated, Blue: downregulated. Selection criteria: log2 |fold change| > 1 and false discovery rate <0.05; (B) Comparison of the differential expression of four tumor proliferation-related genes (*HELLS, ANLN, LAMA1, UBE2C*) between the long-survival benefit and short-survival benefit groups; (C) Comparison of differential expression and its score of hypoxia-associated genes between the long-survival benefit and short-survival benefit groups; (D) Genomic enrichment analysis (GSEA) between long-survival benefit and short-survival benefit groups; (E) GO analysis of differentially expressed gene between the long-survival benefit and short-survival benefit groups.

### 3.5 PPI network of putative proteins

**Fig. 5:**
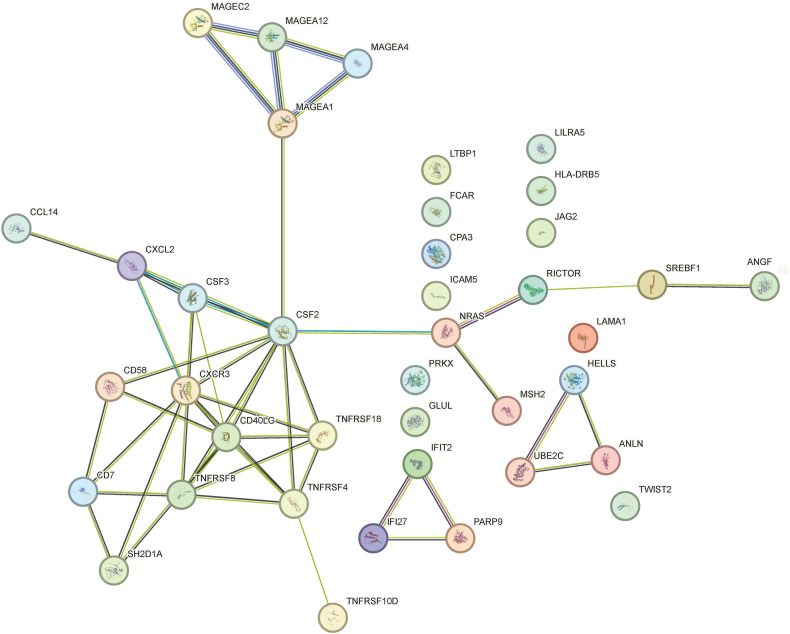
Results of Protein-protein interaction (PPI) network.

To summarize the above DEGs, PPI networks were built using the proteins encoded by these genes implicated (Fig. 5). The results indicate that there is a strong interaction among immune-related genes. Additionally, members of the melanoma-associated antigen (MAGE) family, including MAGEC2, MAGEA12, MAGEA4, and MAGEA1, also exhibit a strong interaction. Currently, MAGEA4 is regarded as a potential target for tumor treatment, and global pharmaceutical companies have developed several drugs targeting this target, such as Afamitresgene Autoleucel and RG6290 [33,34].

## 4 Discussion

Glioblastoma is a malignant brain tumor that is highly aggressive and tends to recur or progress even after undergoing standard first-line treatment [35]. The prognosis for recurrent glioblastoma is very bleak, with a median survival period of only 6 months [36]. There is no consensus on the optimal treatment after disease progression or recurrence. Various novel agents targeting important genes, molecular receptors, or certain regulatory factors associated with glioblastoma cell proliferation, invasion, and angiogenesis have also been clinically evaluated, but with varying efficacy [37,38]. Previous studies have explored efficacy correlations with anti-angiogenic targeted drugs, such as bevacizumab, cilengitide, and enzastaurin, in terms of IDH mutations [39,40], MGMT methylation levels [41], peripheral blood protein molecular expression [42], and TME characters [10] to identify patients that could obtain efficacy benefit. However, the molecular mechanism by which apatinib in combination with temozolomide modulates the clinical benefit of rGBMs has not yet been clarified.

We collected pairs of primary and paired post-treatment recurrence samples from six patients who progressed after standard chemo-radiotherapy treatment to analyze its correlation between tumor-microenvironment interactions. The expression of proliferation-related genes (*MKI67, CEP55, KIF2C*) was significantly lower in the relapse samples. We also found the expression of M1 macrophage and immune response gene signature score FCR were up-regulated in recurrent samples. Chronic inflammation of tumor microenvironment is now widely acknowledged as a key factor contributing to the advancement of glioma [43]. The findings of a previous study indicated that prolonged administration of radiotherapy and temozolomide may exacerbate the immunosuppressive tumor microenvironment in malignant gliomas through upregulating Foxp3 [44]. However, our results on RNA level showed the expression level of FOXP3 didn't increase after treatment of radiotherapy and temozolomide. While our findings suggests that the combination of radiotherapy and TMZ may elicit a certain degree of activation in anti-tumor immunity. Radiation can generally stimulate local tumor immunity and promote anti-tumor immune responses through various molecular mechanisms [45]. Therefore, the impact of standard therapy on the GBM tumor microenvironment may require more research.

Then 22 rGBM who have completed apatinib combined with temozolomide treatment performed RNA expression analysis to explore the likely molecular mechanisms associated with the benefit of apatinib combined with temozolomide treatment from the tumor microenvironment. Patients who responded to apatinib treatment (CR + PR) had higher endothelial (P = 0.0049) and apoptosis (P = 0.0064) scores. Previous basic trials have demonstrated that apatinib can inhibit p-VEGFR expression and the activation of downstream targets in the VEGFR2 signaling pathway. This inhibition resulted in a reduction of glioma cell proliferation and metastasis, as well as enhancement of the anti-tumor activity of temozolomide [9]. A cell assay of non-small cell lung cancer (NSCLC) revealed that apatinib triggered autophagic and apoptotic cell death via VEGFR2/STAT3/PD-L1 and ROS/Nrf2/p62 signaling in lung cancer [46]. Zhang's team has demonstrated that apatinib combined with salidroside significantly reduced hypoxia-inducible factor 1(HIF-1α), vascular endothelial growth factor (VEGF), matrix metallopeptidase (MMPs), and multidrug resistance 1 (MDR1) gene expression, and enhanced the chemosensitivity of gastric cancer cells by regulating the hypoxic microenvironment as well as up-regulating pro-apoptotic gene expression [47]. Furthermore, apatinib plays a crucial role in inhibiting proliferation and growth, as well as promoting apoptosis and inducing cell-cycle arrest and autophagy. This is achieved through regulation of the VEGFR2/STAT3/Bcl-2 signal pathway in both in vivo and in vitro studies of osteosarcoma [48]. Apatinib can also optimize the immunosuppressive tumor microenvironment and improve its anti-tumor effectiveness through various mechanisms. These include enhancing the infiltration of CD8^+^ T cells, promoting Tumor-associated macrophages (TAMs) recruitment, and reducing the levels of transforming growth factor-β (TGF-β) as well as decreasing PD-L1 expression [49]. However, in our study, there were no significantly differential distributions of VEGF related genes (VEGFA, VEGFB and VEGFC) and infiltrating immune cells in different efficacy groups. We did find a modifying role of apatinib combined with temozolomide in regulating tumor proliferation as well as the microenvironment in which the tumor is residing.

In the process of further exploring prognostic markers, we found a convergence of changes in the tumor microenvironment. There were four tumor progression-related genes was deregulated in both of response group and long benefit groups. *HELLS,* lymphoid-specific helicase gene, is associated with gene repair as well as chromosomal stability, and high expression of the *HELLS* gene is associated with glioblastoma progression and poor prognosis [50]. *ANLN* gene encodes an actin-binding protein and regulates mitosis and cytokinesis in gliomas [51]. It has been reported that increased *ANLN* expression promotes cytokinesis and proliferation in cells of esophageal squamous cell carcinoma, and is linked to a negative prognosis in patients with esophageal squamous cell carcinoma (ESCC) [52]. *LAMA1* (Laminin α1) gene was high expression in primary glioblastoma in contrast to normal brain tissue [53], and the over-expression of *LAMA1* was associated with aggressive phenotypes in ESCC, while the high expression of *LAMA1* patients had a shorter PFS or OS than low expression patients [54]. *UBE2C* encodes the protein of ubiquitin conjugating enzyme E2C involved in cell cycle regulation. It has been shown that high expression of this gene is significantly associated with high grade gliomas (WHO 3/4) and reduced overall survival time in glioblastoma [55]. The PPI analysis suggests that HELLS, ANLN, and UBE2C are functionally associated (Fig. 5). Although no current literature reports a direct interaction among these three genes, they are all implicated in the regulation of the cell cycle as well as in the initiation and progression of cancer. Therefore, in the future, we can explore the changes of these three genes in rGBM in a larger population through PCR or immunohistochemistry and other means, and study their interactions and regulatory mechanisms.

There are certain limitations in our study. Firstly, the sample size is extremely limited, which restricts the statistical power. Additionally, we focused exclusively on the RNA level, recognizing that the regulatory mechanism of apatinib is complex and requires multi-dimensional analysis, including proteomics, methylation, and spatial transcriptomics. In addition, the 4.2-month mPFS cutoff value was adopted from the bevacizumab trial and may not fully capture the response dynamics of apatinib in this specific patient cohort.

In conclusion, our study demonstrated that the effective or long survival benefit of apatinib in combination with temozolomide for the treatment of rGBM may be related to the down-regulation of tumor-progression related gene expression, inhibition of tumor cell proliferation, and the modulation of the hypoxia environment as well as the up-regulation of the organic immune response. In the future, large sample size validation and multiple-omics studies are needed.

## Consent to participate

Written informed consent was obtained from each patient.

## Ethics approval

The study was registered on ClinicalTrails (NCT04814329) and approved by the institutional ethics committee of Sanbo Brain Hospital, Capital Medical University (SBNK-YJ-2021-010-02), and informed consent was also obtained from the patients.

## Data availability statement

The data that support the findings of this study are available from the corresponding author, Jun-Ping Zhang doczhjp@mail.ccmu.edu.cn, upon reasonable request.

## Funding information

This work was supported by Scientific Research of Sanbo Brain Hospital Capital Medical University (Grant number: 2021ZZLX03).

## CRediT authorship contribution statement

**Jingjing Ge:** Data curation, Project administration, Writing – original draft, Writing – review & editing. **Cheng Li:** Investigation, Methodology, Writing – review & editing. **Fengjun Xue:** Project administration, Resources, Supervision, Writing – review & editing. **Chi Zhao:** Project administration, Supervision, Writing – review & editing. **Chenchen Kong:** Data curation, Resources, Writing – review & editing. **Shaopei Qi:** Supervision, Validation, Visualization, Writing – review & editing. **Qianqian Duan:** Project administration, Visualization, Writing – original draft, Writing – review & editing. **Qin Zhang:** Data curation, Formal analysis, Software, Visualization, Writing – review & editing. **Junping Zhang:** Conceptualization, Funding acquisition, Project administration, Supervision, Writing – review & editing.

## Declaration of competing interest

The authors have no conflict of interest.

## Figures and Tables

**figs1:**
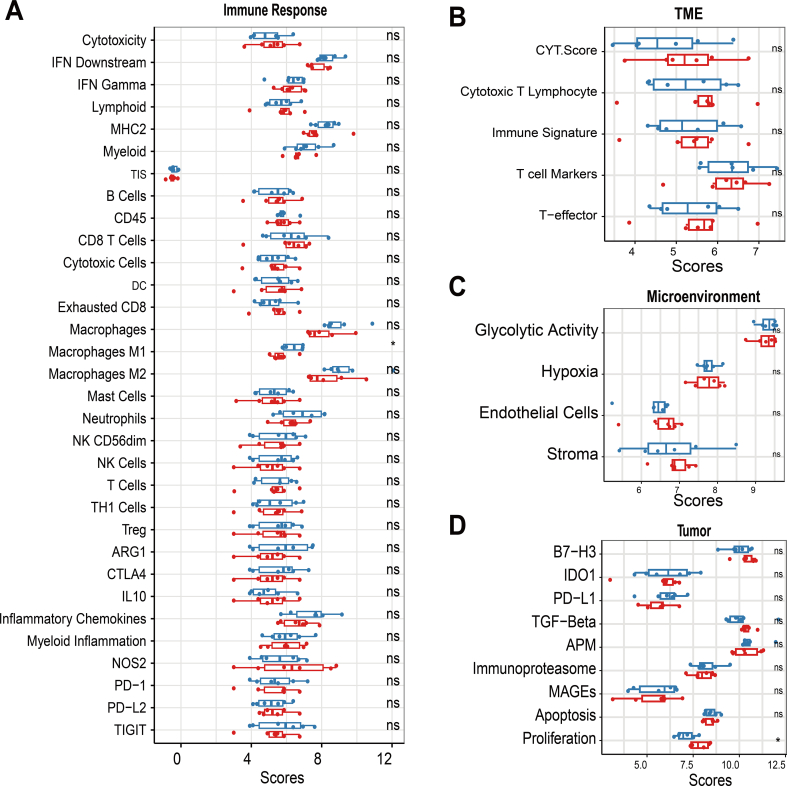


**figs2:**
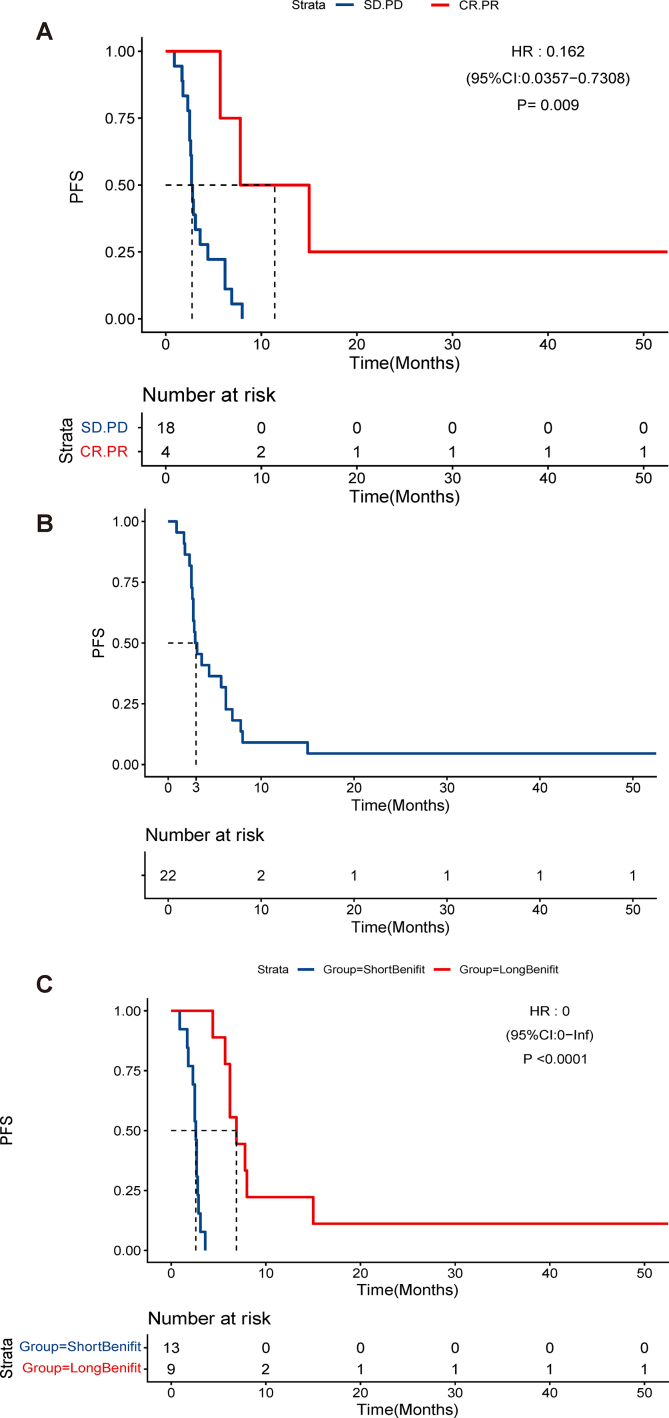


**figs3:**
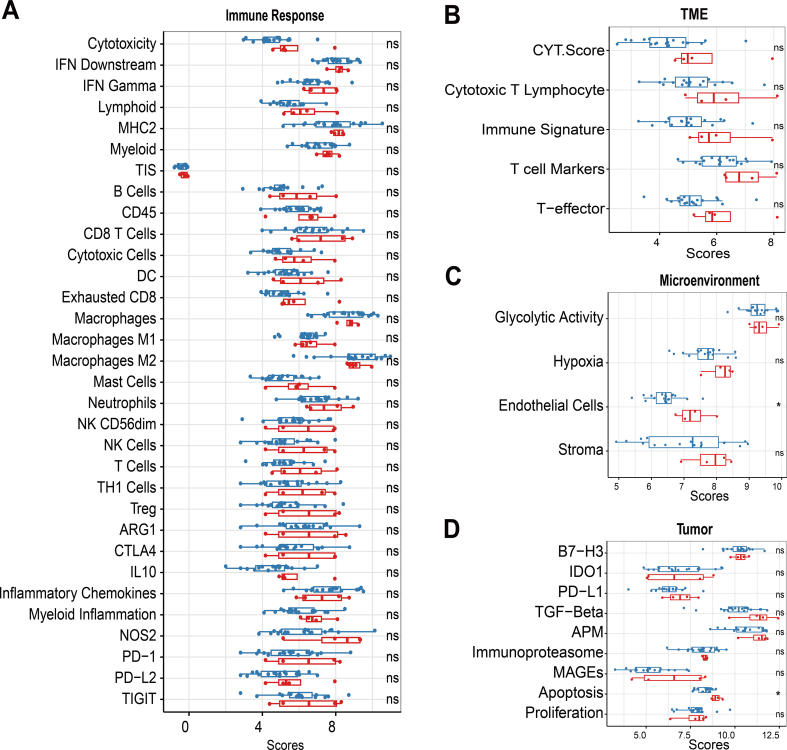


**figs4:**
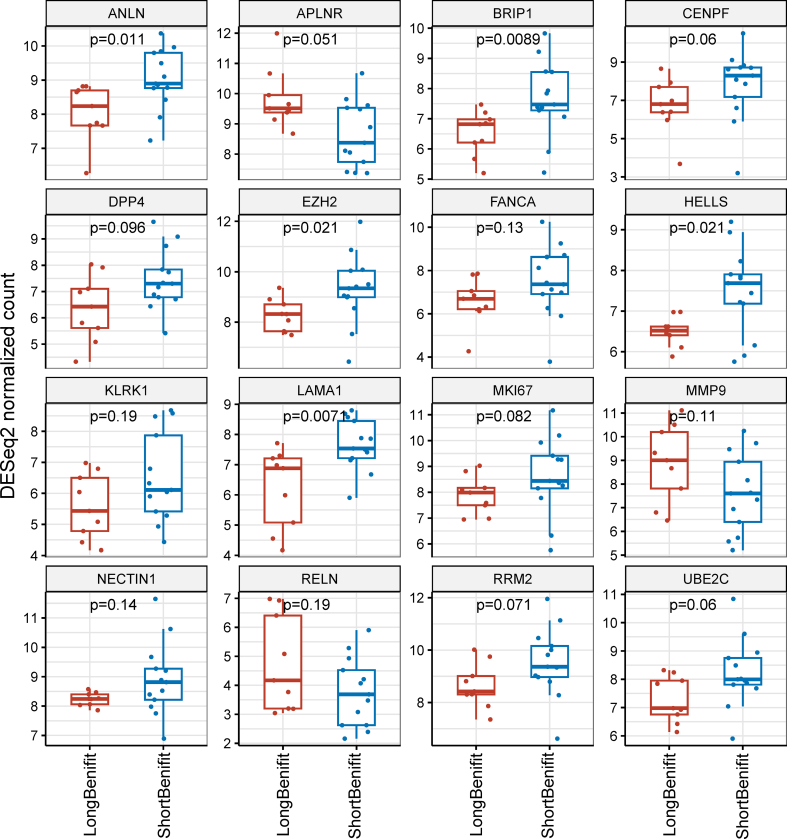


**figs5:**